# Expression profiling of the ubiquitin conjugating enzyme UbcM2 in murine brain reveals modest age-dependent decreases in specific neurons

**DOI:** 10.1186/s12868-015-0194-y

**Published:** 2015-11-13

**Authors:** Chelsea M. Larabee, Constantin Georgescu, Jonathan D. Wren, Scott M. Plafker

**Affiliations:** Aging and Metabolism Research Program, Oklahoma Medical Research Foundation, 825 Northeast 13th Street, Oklahoma City, OK USA; Arthritis and Clinical Immunology Research Program, Oklahoma Medical Research Foundation, 825 Northeast 13th Street, Oklahoma City, OK USA; Oklahoma Center for Neuroscience, University of Oklahoma Health Sciences Center, Oklahoma City, OK USA

**Keywords:** Ubiquitin, UbcM2, Neurons, Hindbrain, Cortex

## Abstract

**Background:**

UbcM2 is a ubiquitin-conjugating enzyme with roles in the turnover of damaged and misfolded proteins, cell cycle progression, development, and regulation of the antioxidant transcription factor, Nrf2. Recent screens have identified binding partners of the enzyme that are associated with various neurodegenerative diseases, and our previous studies have shown that UbcM2 is enriched in retina and brain.

**Results:**

In the current study, we characterized UbcM2 protein expression in various structures and cell types in the murine brain. Immunofluorescence analysis of paraffin-embedded brain sections revealed that UbcM2 is ubiquitously expressed throughout the brain, is enriched in hindbrain and cortex, and is robustly expressed in neurons. In contrast, the enzyme is undetectable in most astrocytes and microglia. As dysfunction of the ubiquitin proteasome system (UPS) has been linked to many age-related neurological diseases, we compared UbcM2 expression levels in young versus aged wild-type mice and found a global decrease in expression in aged brains, with reductions of 10 % or greater in five substructures (cerebellar granule cell layer, primary motor cortex, olfactory nucleus, superior colliculus, and secondary visual cortex).

**Conclusions:**

These studies represent the first protein expression profiling of a ubiquitin-conjugating enzyme in the brain and support the notion that deficits in protein degradation and proteostasis associated with neurodegenerative diseases may be, in part, attributable to age-dependent reductions in the enzymatic machinery of the UPS.

**Electronic supplementary material:**

The online version of this article (doi:10.1186/s12868-015-0194-y) contains supplementary material, which is available to authorized users.

## Background

The ubiquitin (Ub) proteasome system (UPS) is a major intracellular protein degradation pathway. The central protein of this pathway is Ub, a highly conserved 76 amino acid polypeptide that is conjugated to target proteins through a hierarchal enzyme cascade consisting of a Ub-activating enzyme (E1), a Ub-conjugating enzyme (E2), and a Ub ligase (E3). E3s can be single proteins or multi-subunit complexes that recruit and facilitate the transfer of Ub to substrates. Humans have at least two E1s, 36–40 E2s, and up to 1000 E3s [1]. Thus, a single E2 can partner with a host of E3s, and, likewise, a given E3 can engage multiple E2s. Ubiquitylation targets recipient proteins to a number of fates, the best studied of which is degradation by the 26S proteasome. Ubiquitylation has also been shown to regulate protein localization, DNA repair, signal transduction, and a host of other functions (reviewed in [2]). A distinct class of enzymes collectively referred to as de-ubiquitylating enzymes, or DUBs, counter ubiquitylation by removing Ub from substrates. The balance between ubiquitylation and de-ubiquitylation must therefore be finely tuned to maintain proteostasis and cellular fidelity.

Dysregulation of the UPS is associated with many pathologies, including cardiovascular, inflammatory, and neurodegenerative diseases. UPS activity decreases in normal aging as well as in Alzheimer’s disease [3], Parkinson’s disease [4], and age-related macular degeneration [5, 6]. Consequently, a cardinal hallmark of these diseases is the accumulation of toxic protein aggregates; these are often associated with oxidative and proteotoxic stress and damage [7]. In most disorders, it remains to be determined if decreased UPS activity is a primary cause or a secondary consequence of these neurodegenerative pathologies.

A great deal of work has been done to characterize the biochemical mechanisms employed by E2s and E3s, but few studies have focused on cataloging the expression profiles of these enzymes in different organs, tissues, and cell types. In the work reported here, we analyzed the protein expression profile of UbcM2 in the brain. This enzyme is a highly-conserved metazoan E2 that plays roles in proteasome-mediated turnover of proteins [8], cell cycle progression [9, 10], and regulation of the antioxidant transcription factor, Nrf2 [11, 12]. The human and mouse protein sequences of UbcM2 are identical, and the human form is called UBE2E3. Although a comprehensive assessment of the in vivo functions of UbcM2 has not been performed in higher organisms, a functional homolog in yeast has been shown to mediate the degradation of misfolded and oxidatively-damaged proteins [8]. We have previously shown that the enzyme is ubiquitously expressed throughout the body but is enriched in the retina and brain in mice [13]. We further characterized the expression profile in the neuroretina as well as the retinal pigment epithelium [13]. Subsequently, high throughput screens for protein–protein interactions revealed that UbcM2 interacts with the Huntington’s Disease proteins HTT [14] and RNF2 [15] and with the spinocerebellar ataxia-associated protein ATXN1 [16]. More recently, UbcM2 was shown to play a major role in the ubiquitylation status of TDP-43, a DNA-binding protein found in aggregates of amyotrophic lateral sclerosis and subtypes of frontotemporal lobar degeneration [17]. These interactions prompted us to carry out a cell type- and structure-specific immunofluorescence analysis of UbcM2 in mouse brain tissue and to determine if expression of the enzyme is altered in aged mice. We report here the first assessment of the localization and abundance of an E2 in the brain. This study serves as a launching point for the characterization of E2s in both healthy and pathological brains with the hopes of better understanding how UPS deficits contribute to neuropathologies.

## Results

### Validation of the α-UbcM2 antibody

**Fig. 1:**
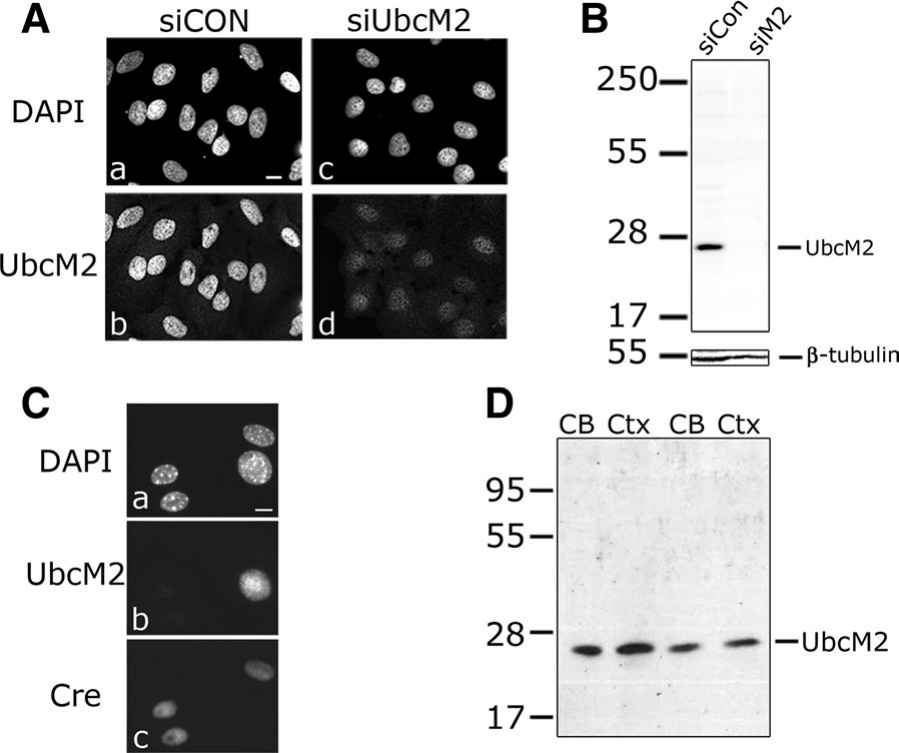
Validation of α-UbcM2 antibody. **A** HeLa cells were treated with 12 pmol of control siCON siRNA (*a*, *b*) or UbcM2-specific siRNA (*c*, *d*) for 72 h before fixation and immunostaining with α-UbcM2 (*b*, *d*). Nuclei were counterstained with DAPI (*a*, *c*). 60×, *size bar* 10 µm. **B** RPE-1 cells were treated with siCON (*left lane*) or siUbcM2 (*right lane*) for 72 h and solubilized for western blot analysis with α-UbcM2. Anti-β-tubulin is used as a loading control. **C** UbcM2^Flox/Flox^ MEFs expressing an inducible Cre recombinase were fixed and labeled with DAPI (*a*), α-UbcM2 (*b*), and α-Cre (*c*). 3 of the 4 cells express Cre and lose UbcM2 expression whereas the non-Cre expressing cell retains UbcM2 expression. **D** Cell lysates prepared from mouse cerebellum (CB) and cortex (Ctx) were analyzed by α-UbcM2 western blot analysis

To determine the protein expression profile of UbcM2 in mouse brain, a rabbit polyclonal antibody raised against the N-terminal 58 residues of UbcM2 was affinity purified and validated in HeLa cells treated with either control siRNA (siCON) or UbcM2-specific siRNA (siUbcM2). 3 days post-siRNA treatment, cells were fixed and immunostained with α-UbcM2 and nuclei were counterstained with DAPI. α-UbcM2 labeling revealed that the enzyme localized to the nucleus (Fig. 1A, panel c), as we reported previously [18, 19], and that the knockdown efficiency was 60–70 % (data not shown). Western blot analysis from human retinal pigment epithelial cell-derived lysates treated with siCON or siUbcM2 shows a single band at the size of UbcM2 (23 kDa) in siCON-treated cells and loss of the band in siUbcM2-treated cells (Fig. 1B). Additional validation of α-UbcM2 came from analyzing mouse embryo fibroblasts (MEFs) harboring floxed alleles of UbcM2 and stably transduced with trimethoprim-inducible Cre recombinase. As shown in Fig. 1C, Cre was induced in three of the four cells in the field and led to ablation of the α-UbcM2 signal in all three cells. The cell lacking Cre expression retained UbcM2 immunolabeling. In cell lysates derived from mouse cerebellum (CB) and cortex (Ctx), α-UbcM2 detected a single band of the predicted migration for the enzyme (Fig. 1D).

### UbcM2 is expressed in neurons and oligodendrocytes of mouse brain

**Fig. 2:**
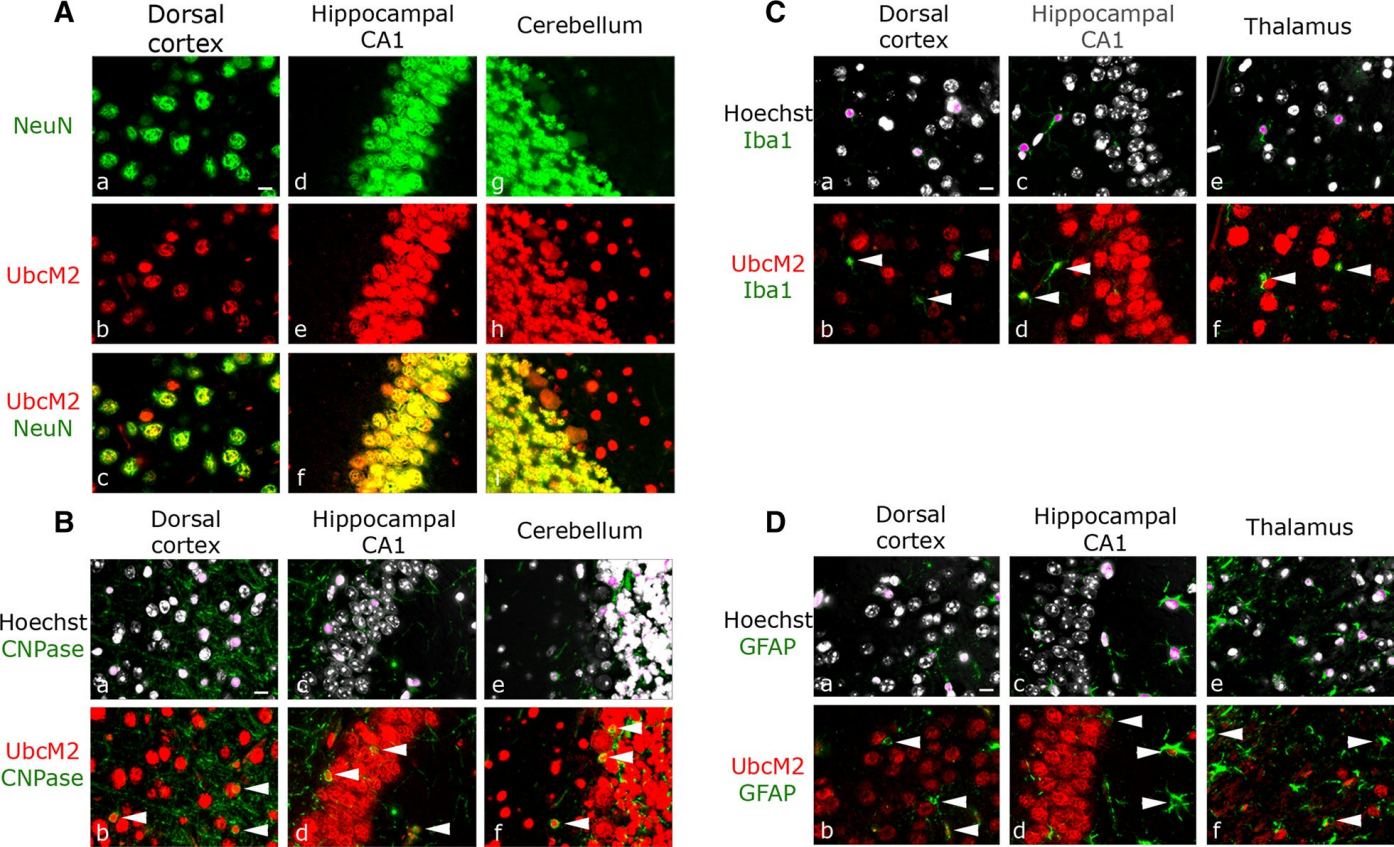
UbcM2 is expressed in neurons and oligodendrocytes but is undetectable in most microglia and astrocytes. Representative images from stained 7 μm, paraffin-embedded sagittal C57BL/6 brain sections of dorsal cortex and CA1, along with cerebellum (**A**, **B**) or thalamus (**C**, **D**). **A** Nuclear neuronal marker NeuN (*green*) and UbcM2 (*red*) with *yellow* indicating colocalization in the merged images (*c*, *f*, *i*). **B**–**D** Co-staining with cell type-specific marker CNPase for oligodendrocytes (**B**), Iba1 for microglia (**C**), or GFAP for astrocytes (**D**) shown in *green* with Hoechst (*white*, *a*, *c*, *e*) to mark nuclei or with UbcM2 (*red*, *b*, *d*, *f*). *White arrowheads* indicate oligodendritic (**B**), microglial (**C**), or astrocytic (**D**) nuclei. 60×, *size bar* 10 µm

We next co-immunolabeled sagittal brain sections with α-UbcM2 and antibodies specific for various cell types resident in brain. Co-staining with an antibody against the nuclear neuronal marker NeuN revealed that the overwhelming majority of UbcM2 is expressed in neurons and that essentially all neurons express the enzyme (Fig. 2A). UbcM2 expression was also consistently observed in CNPase-positive oligodendrocytes throughout brain (Fig. 2B). Within the limits of detection of the UbcM2 antibody, the enzyme was minimally detected or absent in most microglia, labeled by α-Iba1 staining (Fig. 2C), and in astrocytes, labeled by α-GFAP staining (Fig. 2D). Curiously, a subpopulation of microglia and astrocytes displayed robust UbcM2 expression, examples of which are depicted in representative images of hippocampal CA1 sections (white arrows in Fig. 2C, panel d, and Fig. 2D, panel d, respectively). What distinguishes these rare cells from the remainder of glial cells that do not express detectable levels of UbcM2 remains to be determined. Due to the prevalence of UbcM2 in NeuN-positive cells (i.e., neurons), we focused the remainder of this study on quantitatively analyzing the relative expression levels of UbcM2 in neurons.

### UbcM2 is universally expressed in neurons throughout mouse brain

**Fig. 3:**
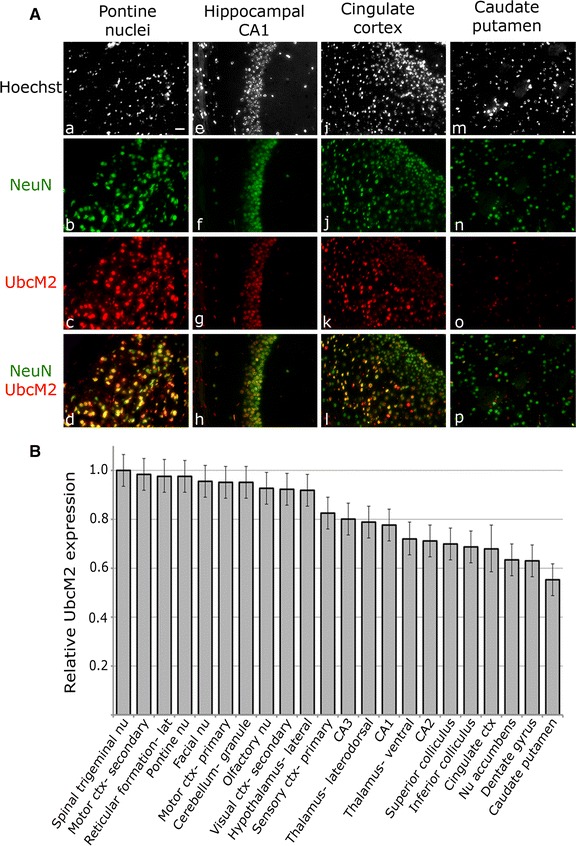
Neurons express UbcM2 at relatively high levels in hindbrain and low levels in caudate putamen. **A** Representative images of 7 μm, paraffin-embedded sagittal brain sections from a 4-month old C57BL/6 mouse. Anatomical regions shown are pontine nuclei (*a*–*d*), hippocampal CA1 (*e*–*h*), cingulate cortex (*i*–*l*), and caudate putamen (*m*–*p*). DNA is counterstained with Hoechst (*white*), and sections are immunostained for the nuclear neuronal marker NeuN (*green*) and UbcM2 (*red*). Co-localization of NeuN and UbcM2 is indicated by *yellow* in the merged images (*d*, *h*, *l*, *p*). 20×, *size bar* 30 μm. **B** Brain substructure-specific UbcM2 expression as measured by fluorescence intensity relative to the area of highest expression (spinal trigeminal nucleus). *Error bars* indicate 95 % confidence intervals. Disjoint confidence interval *bars* identify regions with significantly different UbcM2 levels

Sagittal brain sections were probed with α-UbcM2 and α-NeuN to determine the relative expression of neuronal UbcM2 in 22 anatomically-distinct structures. A mouse brain atlas [20] was used during the sectioning and analysis to ensure correct orientation using white matter, cerebellum, and various structural markers as landmarks. These landmarks also ensured that studies compared the same sagittal plane(s) within each brain. Representative photomicrographs of UbcM2 staining in various substructures (Fig. 3A) demonstrate the range observed throughout the brain, with relatively high expression detected in pontine nuclei (Fig. 3A, panel c), intermediate levels detected in hippocampal CA1 and cingulate cortex (Fig. 3A, panels g and k, respectively), and relatively low levels observed in caudate putamen (Fig. 3A, panel o). Notably, UbcM2 was detected in all substructures surveyed [Fig. 3B; Table 1 (“relative UbcM2 expression” column), and Additional file 1: Figure S1]. Relatively high expression was detected in hindbrain and many cortical areas, whereas hippocampus and several basal ganglia components exhibited lower expression of the enzyme (Fig. 3B, Additional file 1: Figure S1).

**Table 1:** UbcM2 expression is decreased in the neurons of specific substructures of the mouse brain

Brain structure	Relative UbcM2 expression	UbcM2 expression % change in aged mice	P value
CA1	0.776	−11.518	0.0590
CA2	0.711	−9.714	0.1500
CA3	0.801	−7.614	0.1800
Caudate putamen	0.553	−6.471	0.4500
Cerebellum-granule layer	0.951	*−14.530*	*0.0032*
Cingulate ctx	0.679	1.317	0.8900
Dentate gyrus	0.630	1.806	0.8100
Facial nu	0.955	1.957	0.6900
Hypothalamus-lateral	0.919	6.637	0.1800
Inferior colliculus	0.687	−7.692	0.2800
Motor ctx-primary	0.951	*−11.111*	*0.0260*
Motor ctx-secondary	0.984	−5.372	0.2600
Nucleus accumbens	0.634	−7.051	0.3500
Olfactory nu	0.927	*−10.526*	*0.0410*
Pontine nu	0.976	7.083	0.1400
Reticular formation-lateral	0.976	−0.917	0.8500
Sensory ctx-primary	0.825	−10.837	0.0560
Spinal trigeminal nu	1.000	−8.943	0.0600
Superior colliculus	0.699	*−14.535*	*0.0320*
Thalamus-laterodorsal	0.789	−3.247	0.5800
Thalamus-ventral	0.720	−0.847	0.9000
Visual ctx-secondary	0.923	*−11.454*	*0.0290*

The expression levels of UbcM2 in the brains of young and aged mice were measured and compared by average cellular α-UbcM2-derived fluorescence intensity in NeuN-positive nuclei. A 5.5 % global decrease of UbcM2 expression was detected in aged versus their strain-matched, younger counterparts. The italic numbers indicate decreases of UbcM2 expression of 10 % or greater in particular substructures of the brain

### UbcM2 expression is modestly decreased in aged mouse brain

**Fig. 4:**
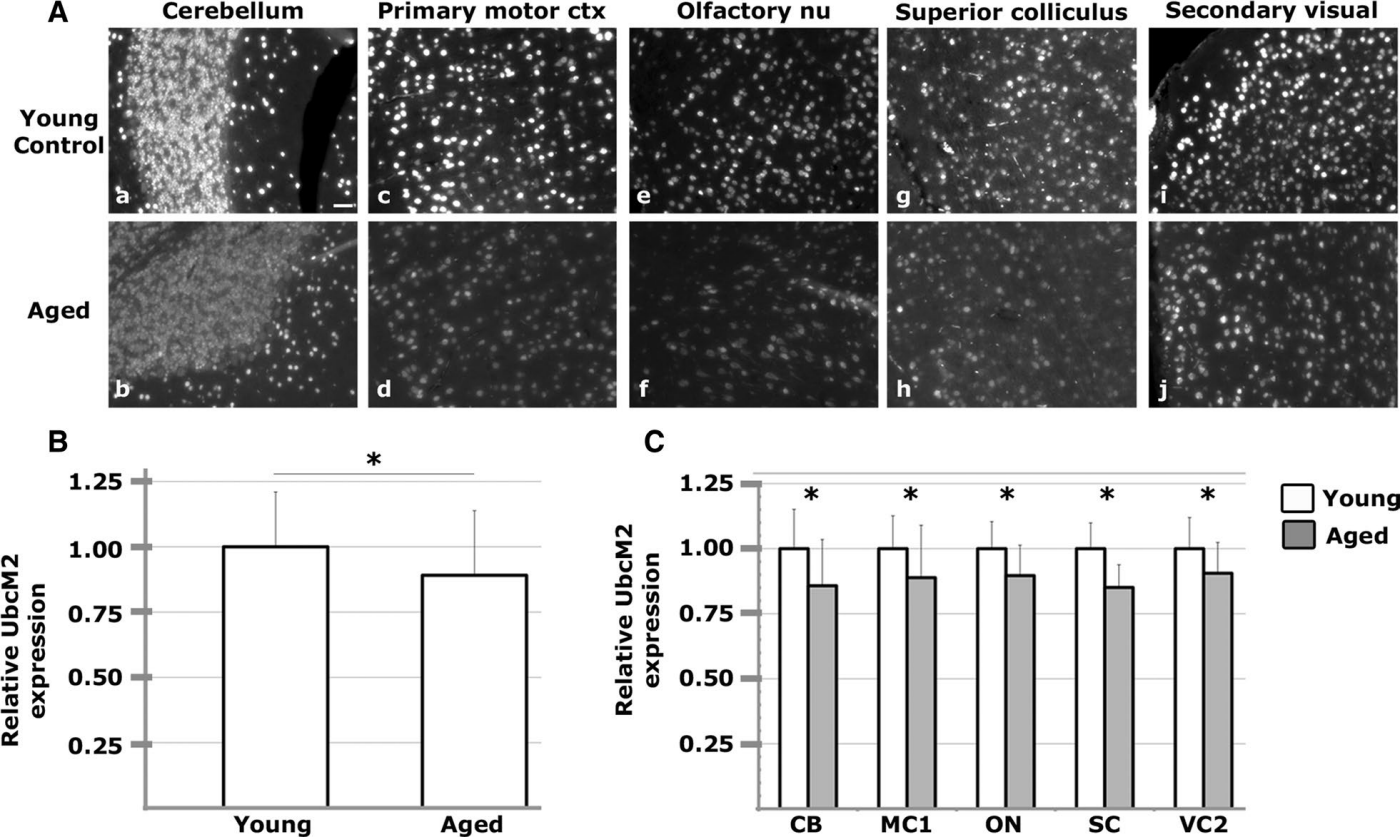
UbcM2 expression is decreased in aged mouse brains. **A** Representative photomicrographs showing decreased UbcM2 expression in aged (*b*, *d*, *f*, *h*, *j*) relative to young control (*a*, *c*, *e*, *g*, *i*) brains in the 5 structures with >10 % differences. 20×, *size bar* 30 μm. **B** Average expression of UbcM2 in whole brain, consisting of the analysis of 22 substructures, indicating a 5.5 % global decrease in aged brain relative to young control. n = 5 in triplicate; p value = 0.046. **C** Average UbcM2 expression in the 5 substructures that exhibited a >10 % decrease. *Asterisks* denote p < 0.05. CB (cerebellum granule cells), MC1 (primary motor cortex), ON (olfactory nucleus), SC (superior colliculus), VC2 (secondary visual cortex)

As the function and efficiency of the UPS decline with age (reviewed in [21, 22]), we next compared the expression levels of UbcM2 in the brains of young and aged mice. Young mice were 1–4 months old, and aged mice ranged from 10–25 months. Sagittal brain sections were examined for neuronal UbcM2 expression as measured by average cellular α-UbcM2-derived fluorescence intensity in NeuN-positive nuclei. An analysis consisting of 22 distinct substructures revealed a modest but statistically significant global decrease of UbcM2 expression of 5.5 % (p value = 0.046) in aged animals as compared to their strain-matched, younger counterparts (Fig. 4A, B; Table 1). Five substructures in particular (cerebellar granule cell layer, primary motor cortex, olfactory nucleus, superior colliculus, and secondary visual cortex) exhibited statistically significant decreases of 10 % or more as a function of age (Fig. 4A, C; Table 1, italic entries).

### UbcM2 expression is unchanged in aged Nrf2-deficient brains relative to aged wildtype

**Fig. 5:**
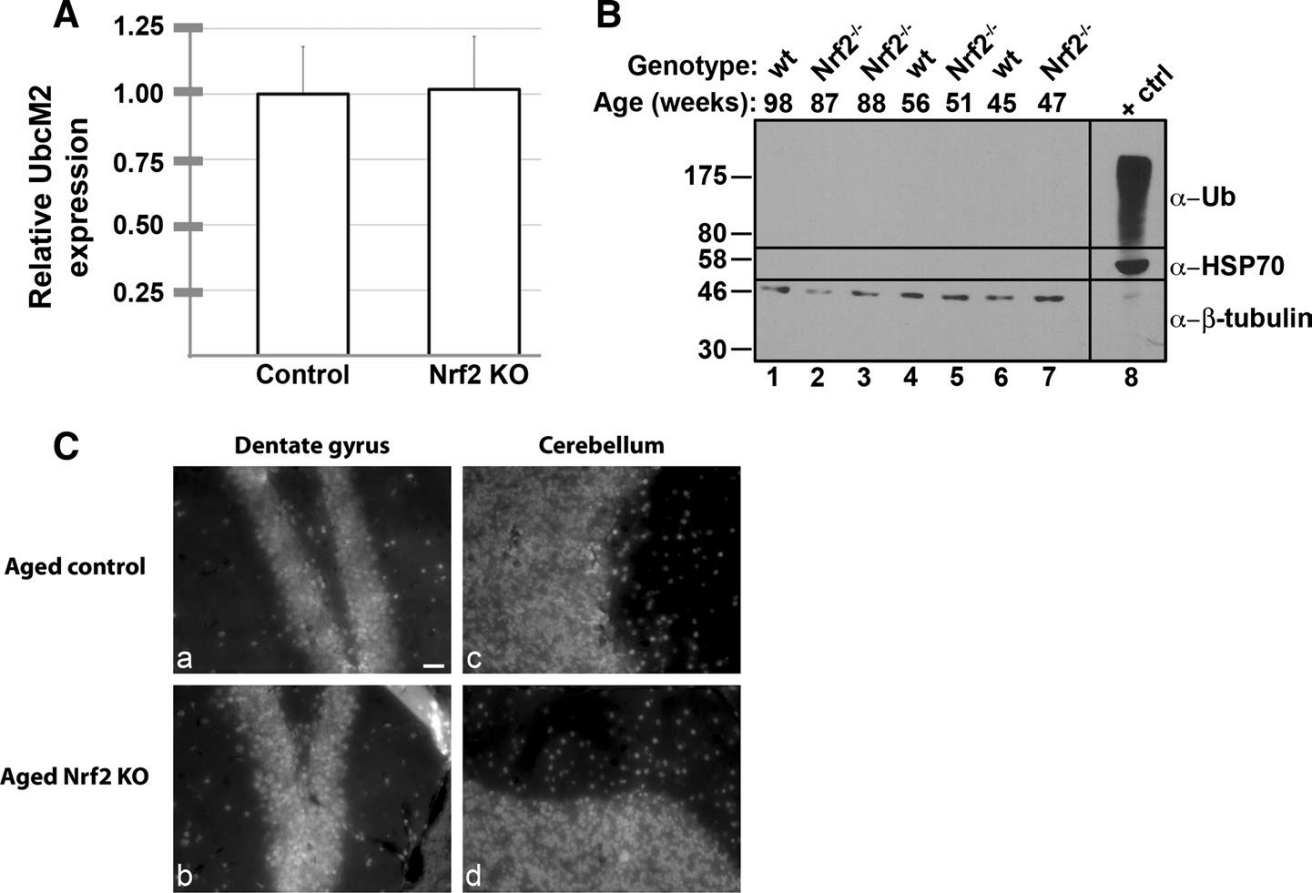
UbcM2 expression is not altered in Nrf2-deficient brains. **A** Average expression of UbcM2 in whole brain, consisting of the analysis of 20 substructures, indicating no change in aged, Nrf2-deficient brain relative to aged, strain-matched controls. n = 5 in triplicate. **B** Western blot analyses of Nrf2 knockout and age/strain-matched wt mice to detect markers of proteotoxicity and stress. Antibodies used are listed to the *right* of each panel, and the age and genotype of the mice are indicated above each *lane*. The positive control (+ctrl) for HSP70 induction and the accumulation of polyubiquitylated proteins is from retinal pigment epithelium (RPE-1) cells treated for 8 h with 10 µm proteasome inhibitor MG132 and harvested with 2 times concentrated Laemmli buffer. The *vertical line* between *lanes 7* and *8* represents a lane in which molecular weight markers were run. The migration of these markers is indicated to the *left*. **C** Representative photomicrographs of dentate gyrus (*a*, *b*) and cerebellum (*c*, *d*) from aged Nrf2-deficient (92 week; *b*, *d*) and strain-matched control (98 week; *a*, *c*) brains immunolabeled with an α-ubiquitin antibody

We have shown that UbcM2 regulates Nrf2 [11, 12], an antioxidant transcription factor that induces the expression of genes encoding cytoprotective proteins and enzymes involved in redox homeostasis and proteostasis [23–25]. Thus, genetic ablation of Nrf2 is predicted to increase the abundance of oxidatively-damaged proteins destined for Ub-mediated degradation. Nonetheless, we did not observe altered levels or expression patterns of UbcM2 in aged cohorts of this knockout strain relative to age-and strain-matched controls (Fig. 5A; Table 2). Consistent with these data, we found by western blotting that the brains of these animals do not accumulate polyUb aggregates or heat shock protein 70 (HSP70), markers of disrupted proteostasis (Fig. 5B), or have increased anti-Ub labeling in paraffin sections (Fig. 5C). These data indicate that the steady-state level of overt proteotoxic stress in Nrf2 knockout brains is relatively minimal. Curiously, these knockout mice reportedly undergo spontaneous retinal degeneration in an age-dependent fashion [26]. We surmise that this phenotype is a function of the retina being exposed to the additional stress derived from constant photo-oxidative challenge throughout the life of the animal.

**Table 2:** UbcM2 expression does not change in aged Nrf2-deficient brains relative to wild type controls

Brain structure	UbcM2 expression % change in Nrf2 KO mice	P value
Amygdala	−1.296	0.83
CA1	−5.181	0.53
CA2	−1.623	0.86
CA3	−4.655	0.54
Caudate putamen	5.078	0.61
Cerebellum-granule	−2.908	0.66
Dentate gyrus	−2.956	0.72
Globus pallidus	6.630	0.37
Inferior colliculus	−5.409	0.77
Motor ctx-primary	4.739	0.42
Nu accumbens	1.304	0.89
Orbital ctx	8.293	0.19
Piriform ctx	5.063	0.40
Sensory ctx-primary	−3.221	0.61
Spinal trigeminal nu	9.589	0.12
Substantia nigra	1.952	0.71
Superior colliculus	9.722	0.27
Thalamus-lateral	−8.556	0.24
Thalamus-ventral	−2.289	0.77
Visual ctx-secondary	7.426	0.26

## Discussion

The principal advance of this work is that it represents the first description of the cell- and structure-specific expression and distribution of a UPS E2 in the mouse brain. Our previous studies to develop a global UbcM2 knockout mouse strain resulted in a failure to produce homozygous knockouts, consistent with UbcM2 loss causing embryonic lethality (S. Plafker, unpublished results) and knockdown of the enzyme inducing a cell cycle arrest/exit [10]. The expression profiling described in the present study is therefore a requisite first step for directing future efforts at genetically ablating the enzyme in specific anatomical structures of the brain. Such knockouts could provide useful models to determine if and how UPS deficits contribute to neurodegeneration.

By immunological staining of paraffin-embedded murine brain sections with a highly specific antibody, we found that UbcM2 is ubiquitously expressed in neurons and oligodendrocytes but is undetectable in the majority of microglia and astrocytes (Fig. 2). These data largely reflect transcriptional profiling data for isolated astrocytes, neurons, and oligodendrocytes showing enrichment of the UbcM2 transcript (referred to by the human gene name, *UBE2E3*), as well as many other components of the UPS, in neurons and oligodendrocytes relative to astrocytes [27]. Approximately half the amount of UbcM2 mRNA was detected in astrocytes relative to neurons, and this level of astrocyte expression is likely due to the fact that the isolated cells were derived from very young mouse brains and cultured several days before analysis. UbcM2 is expressed in replicating cells and is necessary for cell replication [10]. Consistent with this, we hypothesize that the rare population of UbcM2-expressing astrocytes and microglia are likely replicating. Due to the prevalence of UbcM2 in neurons, we quantified the relative expression of the enzyme in NeuN-positive nuclei in 22 brain structures (Fig. 3, Additional file 1: Figure S1; Table 1). We observed relatively robust UbcM2 expression in hindbrain and cortical structures and lower expression in hippocampus and several basal ganglia components. As UPS dysfunction has been linked to multiple age-associated neuropathologies, we also examined whether UbcM2 expression levels change as a function of age. We observed a modest 5.5 % global decrease in neuronal UbcM2 expression in aged brain with a more marked decrease (>10 %) in 5 substructures, namely cerebellar granule cell layer, primary motor cortex, olfactory nucleus, superior colliculus, and secondary visual cortex (Fig. 4; Table 1).

Our results are largely consistent with a qRT-PCR analysis of UbcM2 mRNA localization in mouse embryo [28]. Similar to our findings of protein expression in adult mice [13], UbcM2 mRNA was enriched in embryonic brain with lowest expression in heart and intermediate levels in lung, liver, and kidney. Within embryonic brain, high mRNA levels were detected in cortex and hindbrain, but unlike UbcM2 protein expression, the mRNA was also enriched in thalamus, midbrain, and hippocampus at various stages of embryonic development. These differences may be attributable to changes in UbcM2 expression during development versus post-development and/or a discrepancy between protein and mRNA levels due to translational or degradative regulation.

Although genetic mutations of UbcM2 have not been linked to neurodegeneration to date, the enzyme is highly enriched in numerous brain areas devoted to motor output, including primary and secondary motor cortices, reticular formation, pontine nuclei, facial nucleus, and cerebellum. These data fit with the notion that UbcM2 may contribute to motor function and thereby potentially play a role in motor deficits that characterize a number of neurodegenerative diseases. Notably, GWAS data indicate that UbcM2 can interact with ATXN1 [16], a protein linked to spinocerebellar ataxia (SCA). This condition is marked by motor deficits resulting from cerebellar degeneration, dysfunction of both the UPS and the mitochondria, and an accumulation of Ub-positive aggregates [29]. Interestingly, UbcM2 is enriched in the cerebellum and appears to decline with age (Fig. 4A; Table 1). Moreover, UbcM2 has been shown to ubiquitylate TDP-43 [17], a protein involved in amyotrophic lateral sclerosis (ALS). ALS is a motor disease that can result from degeneration of upper motor neurons located in the motor cortex or reticular formation of the brainstem, both of which express relatively high levels of UbcM2 (Fig. 3B; Table 1). These interactions potentially link the enzyme to the neurodegenerative motor disorders of SCA and ALS. UbcM2 could also be linked to non-pathological, age-related motor deficits as the enzyme is abundant in the cortico-ponto-cerebellar tract, an important motor pathway, and decreases in motor cortex and cerebellar granule cells with age. Both of these structures degenerate in normal aging and contribute to age-related motor dysfunction [30].

## Conclusions

In conclusion, we have performed the first protein expression profiling of a Ub-conjugating enzyme in the mouse brain. We show that the class III E2 UbcM2 is ubiquitously expressed in neurons and oligodendrocytes but is not prevalent in microglia or astrocytes. By quantifying relative α-UbcM2-derived fluorescence from immunohistochemically stained mouse brain sections, we have determined the relative structure-specific neuronal expression of the enzyme. Further, we have observed a modest decrease in neuronal UbcM2 expression in particular sub-structures of the aged mouse brain.

## Methods

### Antibodies

α-UbcM2 was raised in rabbits against a recombinant His_6_-S-tagged polypeptide corresponding to the unique N-terminal 58 residues of the enzyme (MSSDRQRSDDESPSTSSGSSDADQRDPAAPEPEEQ EERKPSATQQKKNTKLSSKTTAK), as previously described [12]. Commercially-available, validated antibodies used throughout these immunofluorescence studies include: mouse polyclonal α-GFAP (1:500; Neuromics MO22136), mouse monoclonal α-myelin CNPase (1:2500; Covance SMI-91R), mouse monoclonal α-Iba1 (1:200; EMD Millipore MABN92), mouse monoclonal α-NeuN (1:2000; EMD Millipore MAB377), mouse monoclonal α-ubiquitin (1:500; Santa Cruz sc-8017), and mouse monoclonal α-cre recombinase (1:2000; Millipore MAB3120). Secondary antibodies used were Alexa_546nm_ Fluor-conjugated goat α-rabbit IgG and Alexa_488nm_ Fluor-conjugated goat α-mouse IgG (1:1000, Molecular Probes). Antibodies used for western blotting include mouse monoclonal α-HSP70 (1:1000; Enzo, Inc ADI-SPA-810), mouse monoclonal α-Ub (1:1000; Santa Cruz sc-8017), and mouse monoclonal anti-β-tubulin (1:1000; Sigma Aldrich T4026). Details for each are listed in Table 3.

**Table 3:** Antibodies used in this study

Antibody	Immunogen	Manufacturer, host, mono-/poly-clonal, catalog number, RRID	Conc. used
Anti-UbcM2	Recombinant His_6_-S-tagged unique N-terminal extension (MSSDRQRSDDESPSTSSGSSDADQRDPAAPEPEEQEERKPSATQQKKNTKLSSKTTAK)	In-house, rabbit, polyclonal	1:500 (IHC)/1:1000 (WB)
Anti-Cre recombinase	aa 77-343 fusion protein	EMD Millipore, mouse, monoclonal, MAB3120, RRID:AB_2085748	1:2000
Anti-GFAP	Recombinant GFAP, boosts with GFAP purified from bovine spinal cord	Neuromics, mouse, polyclonal, MO22136, RRID:AB_2341541	1:500
Anti-myelin CNPase	Purified human myelin CNPase	Covance, mouse, monoclonal, SMI 91, RRID:AB_10120658	1:2500
Anti-Iba1	Linear peptide corresponding to human Iba1/AIF1	EMD Milipore, mouse, monoclonal, MABN92, RRID:AB_10917271	1:200
Anti-NeuN	Purified cell nuclei from mouse brain	EMD Millipore, mouse, monoclonal, MAB377, RRID:AB_2298772	1:2000
Anti-HSP70	Native human HSP70	Enzo, mouse, monoclonal, ADI-SPA-810, RRID:AB_10616513	1:1000
Anti-ubiquitin	Amino acids 1-76 representing full length ub of bovine origin	Santa Cruz Biotechnology, mouse, monoclonal, sc-8017, RRID:AB_628423	1:1000 (WB)/1:500 (IHC)
Anti-β-tubulin	Tubulin from rat brain	Sigma-Aldrich, mouse, monoclonal, T4026, RRID:AB_477577	1:1000

### Cell culture, siRNA transfections, and immunofluorescence

UbcM2-specific siRNA or control siCON siRNA (Dharmacon, Inc.) were combined with INTERFERin (Polyplus transfection) for 10 min at room temperature before being added to 35,000 HeLa or hTERT RPE-1 (cat # CRL-4000; ATCC) cells seeded in 12-well dishes on glass coverslips. A final concentration of 10 nM siRNA was added to the cells in DMEM (cat. # 10-013-CV; Mediatech, Inc.) supplemented with 10 % fetal calf serum (FCS), 100 units/mL penicillin, and 0.1 mg/ml streptomycin sulfate. 72 h post-transfection, cells were fixed in 3.7 % formaldehyde in PBS for 15 min at room temperature and permeabilized in 0.2 % Triton X-100 in PBS for 10 min on ice. Cells were then incubated in blocking solution consisting of 3 % bovine serum albumin (BSA) in PBS for 3 h before being incubated in α-UbcM2 diluted at 1:500 in blocking solution for 1 h at room temperature. Cells were subsequently washed in PBS, incubated in secondary antibody plus 4′,6-diamidino-2-phenylindole (DAPI; 0.1 μg/mL) in blocking solution for 1 h, washed again, and mounted on glass slides.

To generate MEFs, pregnant UbcM2^Flox/Flox^ mice were sacrificed 13.5 days post-coitus and embryos removed by manual dissection from amniotic sacs, washed with ethanol, and the limbs, tail, red organs, and head above the eyes were removed. The remaining material was minced with a sterile razor blade in 4.5 g/L glucose-containing DMEM containing penicillin and streptomycin and trypsinized for 10 min at 37 °C. Non-soluble material was removed by centrifugation, and the remaining cells were cultured in 4.5 g/L glucose-containing DMEM containing penicillin, streptomycin, and 10 % FCS. MEFs were subsequently transduced with a lentivirus expressing a trimethoprim-inducible Cre recombinase, and Cre expression was induced by adding 10 µM trimethoprim to the media for at least 24 h.

### Mouse brain tissue preparation

All animal care was performed in compliance with The Oklahoma Medical Research Foundation Institutional Animal Care and Use Committee-approved protocol. Nrf2^−/−^ mice (10–25 months) were backcrossed 10 generations into a C57BL6 background and have been previously described [26, 31]. C57BL/6 mice were used in the control (1–4 months) versus aged (10–25 months) analysis. Mice were euthanized by CO_2_ asphyxiation and decapitated to remove brains. Tissue was fixed in 10 % neutral buffered formalin at 4 °C overnight then processed and paraffinized using an overnight protocol with ethanol, xylene, and paraffin equilibration in a Shandon Excelsior tissue processor (Thermo Scientific). Brains were cut into 7 μm-thick sagittal sections using a Leica Instruments Microtome (Model 2045 Multicut), mounted on charged glass slides, and warmed on a Lab-Line Instruments slide warmer (Model 26020) overnight. A mouse brain atlas [20] was used throughout the sectioning process to ensure correct orientation using white matter, cerebellum, and various structural markers as landmarks. These same landmarks were observed to ensure studies compared the same sagittal plane within each brain, although we observed very similar UbcM2 expression levels in various planes of the same substructures within a brain (data not shown).

### Immunohistochemistry

Slides were deparaffinized by washes in Histo-Clear (National Diagnostics) followed by serial washes in decreasing amounts of ethanol and then water. Heat-induced epitope retrieval was performed using a Retriever 2100 (Electron Microscopy Sciences) in the accompanying R-buffer AG, and slides were allowed to cool in the retriever for 4–20 h. Slides were then washed in PBS and incubated in blocking solution consisting of 10 % goat serum and 3 % BSA in PBS for 1–3 h at room temperature. Sections were incubated in α-UbcM2 (1:500) or α-Ubiquitin (1:500) and a cell-type specific antibody in blocking solution at 4 °C overnight. The following day, the slides were washed in PBS and incubated in secondary antibody and DAPI at 0.1 μg/mL or Hoechst33342 at 1 μg/mL diluted in 3 % BSA in PBS for 1 h at room temperature in the dark. After a final PBS wash, slides were mounted using ProLong Gold (Cell Signaling Technology) and examined with a Nikon 80i microscope and DXM1200C camera. Images were captured using NIS-Elements software (Nikon), and representative images were processed in Adobe Photoshop (Version 8.0). Of note, all sections quantitatively compared corresponded to a similar parasagittal plane of the brain, were processed at the same time in parallel with the same diluted reagents, and were imaged using identical gain, exposure, and binning settings.

### Western blotting

Brain lysates were prepared from 5 month-old female C57BL/6 mice that were euthanized by CO_2_ asphyxiation and decapitated to remove brains for dissection of cerebellum and cortex on a cold block. Structures were lysed in a buffer consisting of 150 mM NaCl, 20 mM Tris [pH 8], 1 % Triton X-100, 2 mM EDTA, and 1 tablet phosphatase inhibitor (Thermoscientific PI 88266), homogenized with a pestle, and incubated on ice 1–2 h before sonication. Debris was removed by centrifugation, and supernatants collected for analysis. Brain or cell culture lysates were boiled in 2 times concentrated (2X) Laemmli solubilizing buffer (100 mM Tris [pH 6.8], 2 % SDS, 0.008 % bromophenol blue, 2 % 2-mercaptoethanol, 26.3 % glycerol, and 0.001 % Pyrinin Y), resolved by SDS-PAGE and transferred to nitrocellulose. Blots were blocked in 5 % nonfat milk/TBST (0.1 % Tween-20 in Tris-buffered saline) before incubation with primary antibodies. All primary antibodies were diluted 1:1000 in 5 % nonfat milk/TBST. Horseradish peroxidase (HRP)-conjugated secondary antibodies were diluted in 5 % Milk/TBST, and blots were processed with enhanced chemiluminescence.

### Neuronal UbcM2 expression analysis

For each genotype or age comparison, 5 animals were used per group as biological replicates, and per animal, 3 sequential sections on 1 slide were analyzed as technical replicates such that a total of 15 brain sections from 5 animals represent each genotype/age group. A separate 20× picture was taken of each substructure analyzed, and all cells (50–150 nuclei) were evaluated in each image. Only nuclear expression was observed and quantitated, as this study and our previous work demonstrate that UbcM2 is exclusively expressed in the nucleus [13]. UbcM2 expression was measured in terms of α-UbcM2-derived fluorescent intensity in NeuN-positive nuclei using NIH ImageJ software. Separate grayscale pictures were taken for each of the three channels corresponding to nuclei, NeuN, and UbcM2. In ImageJ, each raw NeuN image was subjected to a user-defined threshold such that only NeuN-positive areas were represented. The fluorescence intensity of each NeuN-positive cell was then measured in the corresponding raw UbcM2 image on a scale of 0–255 pixels. Each nuclear UbcM2 measurement was normalized to the background intensity of that image with background defined as an average value of 10 separate measurements of visually UbcM2-negative areas.

### Statistical analysis

In each of the two comparative studies, namely young vs old and Nrf2 knockout vs wildtype, the enzyme level dependence on the covariates was analyzed by fitting a Linear Mixed Effects Model implemented as the lme function in the nlme R package. This function is essentially an extended version of the classical linear regression but is able to accommodate complex data collection design features such as nested layers and within-group correlation. The method formulation [32], computational method [33], and implementation of the model in R [34] have previously been described in detail. As described in the previous section, data collection design involved 3 embedded layers for each group (where group refers to old, young, Nrf2 knockout, or Nrf2 strain-matched wildtype control) as 50–150 measurements (cells) per 22 (aged study) or 20 (Nrf2 study) substructures were analyzed in three sequential brain sections per mouse. Accordingly, the data was analyzed by performing an unequal sample sizes multi-level nested lme where protein level dependence on the fixed effects, namely variable (age or genotype), brain structure, and their interactions, was analyzed with the embedded data collection structure designated as a random effect represented as Mouse/Replicate/Structure. Significance of UbcM2 expression changes in whole brain was assessed with the lme version of the ANOVA function applied on the lme object. Fixed effect coefficient significance tables were extracted with the tTable method while confidence intervals were returned by the intervals function in the nlme package (Tables 1, 2; Figs. 3B, 4B, C, 5A). Slight testing power boosting was achieved by using one-sided tests with the null hypothesis matching the expectation that UbcM2 levels are lower in old mice.

## Additional file

10.1186/s12868-015-0194-y UbcM2 is ubiquitously expressed in neurons of mouse brain. Representative photomicrographs from a 7 µm paraffin-embedded sagittal brain section from a 4-month old C57BL/6 mouse. Nuclei are counterstained with Hoechst (white, panels a, e, i, m), and immunostaining is shown for neuronal nuclear marker NeuN (green, panels b, f, j, n) and UbcM2 (red, panels c, g, k, o). Neuronal UbcM2 expression is represented as yellow in the NeuN and UbcM2 merged images (panels d, h, l, p). A) Hippocampus: CA1 (panels a-d), CA2 (panels e-h), CA3 (panels i-l), and dentate gyrus (panels m-p). B) Hindbrain: cerebellum (panels a-d), facial nucleus (panels e-h), lateral reticular formation (panels i-l), pontine nuclei (panels m-p), and spinal trigeminal nucleus (panels q-t). C) Cortex: cingulate cortex (panels a-d), primary motor cortex (panels e-h), secondary motor cortex (panels i-l), primary sensory cortex (panels m-p), and secondary visual cortex (panels q-t). D) Midbrain: inferior colliculus (panels a-d), superior colliculus (panels e-h), laterodorsal thalamus (panels i-l), and ventral anterior thalamus (panels m-p). E) Ventrorostral: caudate putamen (panels a-d), lateral hypothalamus (panels e-h), nucleus accumbens (panels i-l), and olfactory nucleus (panels m-p). 20X, size bars = 30 μm.

## Abbreviations

UPS: ubiquitin proteasome system
Ub: ubiquitin
E1: ubiquitin activating enzyme
E2: ubiquitin conjugating enzyme
E3: ubiquitin protein ligase
siCON: siRNA control
siUbcM2: UbcM2-specific siRNA
DAPI: 4′,6-diamidino-2-phenylindole
SCA: spinocerebellar ataxia
ALS: amyotrophic lateral sclerosis
