# Supplementary figures and images for: Expression profiling of the ubiquitin conjugating enzyme UbcM2 in murine brain reveals modest age-dependent decreases in specific neurons
Source: BMC Neurosci. 2015 Nov 13;16:76. doi: 10.1186/s12868-015-0194-y (PMC4644300; doi:10.1186/s12868-015-0194-y)

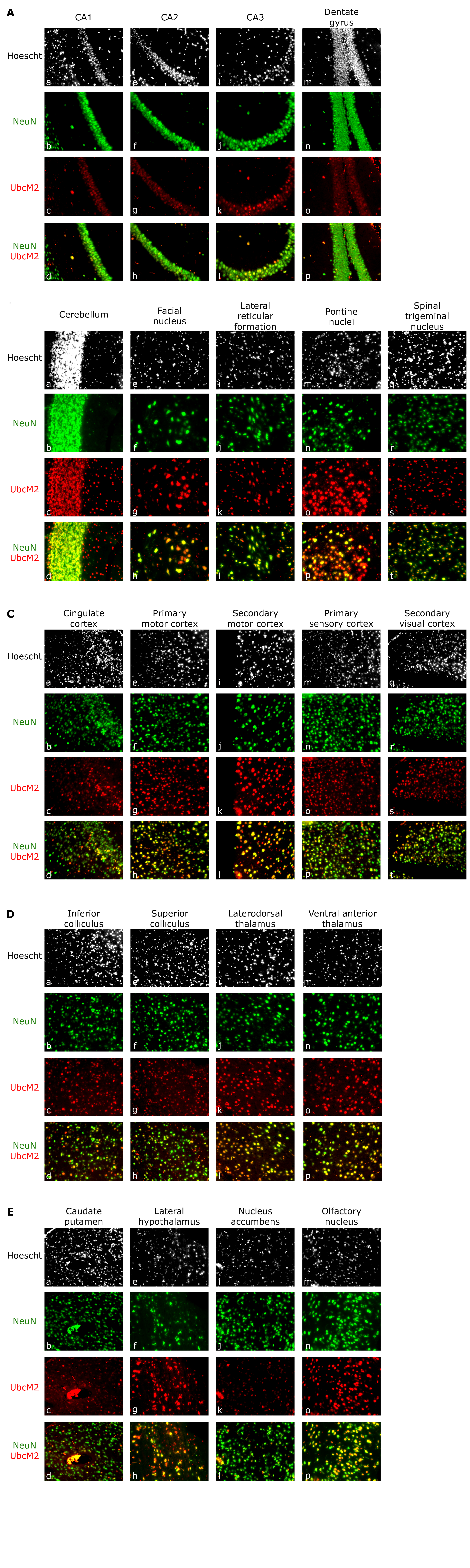

Supplement: Supplementary file 1 — 10.1186/s12868-015-0194-y UbcM2 is ubiquitously expressed in neurons of mouse brain. Representative photomicrographs from a 7 µm paraffin-embedded sagittal brain section from a 4-month old C57BL/6 mouse. Nuclei are counterstained with Hoechst (white, panels a, e, i, m), and immunostaining is shown for neuronal nuclear marker NeuN (green, panels b, f, j, n) and UbcM2 (red, panels c, g, k, o). Neuronal UbcM2 expression is represented as yellow in the NeuN and UbcM2 merged images (panels d, h, l, p). A) Hippocampus: CA1 (panels a-d), CA2 (panels e-h), CA3 (panels i-l), and dentate gyrus (panels m-p). B) Hindbrain: cerebellum (panels a-d), facial nucleus (panels e-h), lateral reticular formation (panels i-l), pontine nuclei (panels m-p), and spinal trigeminal nucleus (panels q-t). C) Cortex: cingulate cortex (panels a-d), primary motor cortex (panels e-h), secondary motor cortex (panels i-l), primary sensory cortex (panels m-p), and secondary visual cortex (panels q-t). D) Midbrain: inferior colliculus (panels a-d), superior colliculus (panels e-h), laterodorsal thalamus (panels i-l), and ventral anterior thalamus (panels m-p). E) Ventrorostral: caudate putamen (panels a-d), lateral hypothalamus (panels e-h), nucleus accumbens (panels i-l), and olfactory nucleus (panels m-p). 20X, size bars = 30 μm. [file 12868_2015_194_MOESM1_ESM.tiff]
